# Analyzing the Effects of Parameters for Tremor Modulation via Phase-Locked Electrical Stimulation on a Peripheral Nerve

**DOI:** 10.3390/jpm12010076

**Published:** 2022-01-08

**Authors:** Jeonghee Kim, Thomas Wichmann, Omer T. Inan, Stephen P. DeWeerth

**Affiliations:** 1 Department of Engineering Technology and Industrial Distribution, Texas A&M University, College Station, TX 77843, USA; 2 Department of Multidisciplinary Engineering, Texas A&M University, College Station, TX 77843, USA; 3 Department of Electrical and Computer Engineering, Texas A&M University, College Station, TX 77843, USA; 4 Department of Neurology, Emory University, Atlanta, GA 30322, USA; twichma@emory.edu; 5 Neuropharmacology and Neurologic Diseases, Yerkes National Primate Research Center at Emory University, Atlanta, GA 30329, USA; 6 School of Electrical and Computer Engineering, Georgia Institute of Technology, Atlanta, GA 30332, USA; omer.inan@ece.gatech.edu (O.T.I.); steve.deweerth@lehigh.edu (S.P.D.); 7 Department of Biomedical Engineering, Lehigh University, Bethlehem, PA 18015, USA

**Keywords:** essential tremor, peripheral nerve stimulation, neuromodulation, parameter optimization, tremor quantification, wearable non-invasive stimulation

## Abstract

(1) Background: Non-invasive neuromodulation is a promising alternative to medication or deep-brain stimulation treatment for Parkinson’s Disease or essential tremor. In previous work, we developed and tested a wearable system that modulates tremor via the non-invasive, electrical stimulation of peripheral nerves. In this article, we examine the proper range and the effects of various stimulation parameters for phase-locked stimulation. (2) Methods: We recruited nine participants with essential tremor. The subjects performed a bean-transfer task that mimics an eating activity to elicit kinetic tremor while using the wearable stimulation system. We examined the effects of stimulation with a fixed duty cycle, at different stimulation amplitudes and frequencies. The epochs of stimulation were locked to one of four phase positions of ongoing tremor, as measured with an accelerometer. We analyzed stimulation-evoked changes of the frequency and amplitude of tremor. (3) Results: We found that the higher tremor amplitude group experienced a higher rate of tremor power reduction (up to 65%) with a higher amplitude of stimulation when the stimulation was applied at the ±peak of tremor phase. (4) Conclusions: The stimulation parameter can be adjusted to optimize tremor reduction, and this study lays the foundation for future large-scale parameter optimization experiments for personalized peripheral nerve stimulation.

## 1. Introduction

Tremor in neurological disorders such as essential tremor (ET) and Parkinson’s disease (PD) [1,2,3,4] can significantly reduce quality of life for patients [5,6,7]. This is particularly true for ET, a chronic and progressive neurological disorder. ET typically occurs with movement, with tremor frequencies of 4–12 Hz [8,9,10,11,12]. This disorder affects about ten million people in the United States. Although tremor movements can be observed in all parts of the body, such as the hands, the head, the voice, the legs, and the trunk, 90% of ET patients experience arm tremor. Symptoms of ET most commonly appear in people in their 40s and older. Nine percent of people over the age of 60 experience tremor [13,14].

Once patients are diagnosed with ET, the general treatment of their symptoms is pharmacotherapy. Common medications for the disorder are primidone and beta receptor blockers (e.g., propranolol) [15,16,17,18,19]. These medications often help, but 40% of patients do not derive meaningful benefits, and some experience significant side effects (e.g., fatigue, nausea, dizziness, sedation) [18,19]. Another possible therapy is the intramuscular injection of botulinum toxin, specifically in tremors with a dystonic component [20,21,22]. Tremor patients also have the option of undergoing deep brain stimulation (DBS) therapy [23,24,25,26,27,28,29] in which a series of electrodes are implanted into the ventral intermediate nucleus of the thalamus. While effective in most treated patients, the cost of this treatment is high, and the prospect of brain surgery is unattractive for many patients.

Tremor can also be suppressed by functional electrical stimulation (FES), which aims to stimulate arm muscles of the upper limbs [30,31,32,33,34,35,36]. FES attenuates tremor in patients with ET or PD. FES at motor threshold, evoking contractions of flexor and extensor muscles either out-of-phase or in phase (co-contraction), modulates tremor. Yet another approach entails the stimulation of afferent pathways [36,37,38,39,40,41,42,43,44,45,46,47,48]. In this case, sub-threshold electrical stimulation of afferent pathways is used [37,38,39,40,41,42,43,44,45,46,47]. It is possible that this type of stimulation modulates neurological pathways that produce tremor. Among these studies [48,49,50], a few (including ours [47]) entailed the use of a wearable setup that applies electrical stimulation to afferent pathways while collecting tremor movements via a motion sensor. One wearable device is commercially available [51] and has been used successfully. The developers of the device did not use real-time parameter adjustment strategies to maximize tremor suppression.

**Figure 1:**
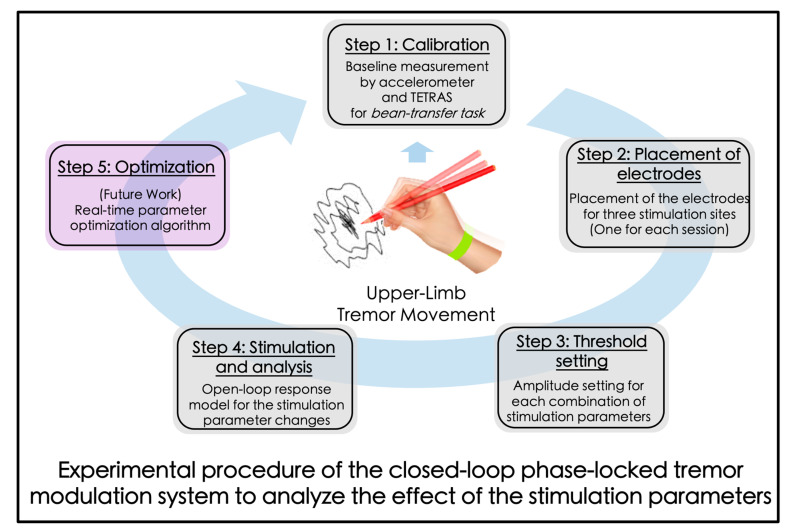
Experimental procedure for the analysis of the effects of the stimulation parameters using the tremor monitor and modulation system.

In a previous publication [47], we showed that external electrical stimulation of the radial nerve, administered via a small wearable device, can significantly reduce tremor in ET participants. The prior study analyzed a limited number of combinations of electrical stimulation parameters in an open-loop configuration. As the first step towards development of a closed-loop (self-adjusting) wearable tremor modulation system, we contrasted several varying parameter combinations (differing in amplitude, frequency, and phase alignment to ongoing tremor) in the current study. The overall concept of the closed-loop stimulation parameter optimization for the tremor modulation via peripheral nerve electrical stimulation is shown in Figure 1. Steps 1–4 of the Figure 1 show the overall experimental procedure of this study.

## 2. Materials and Methods

### 2.1. Experimental Design

**Figure 2:**
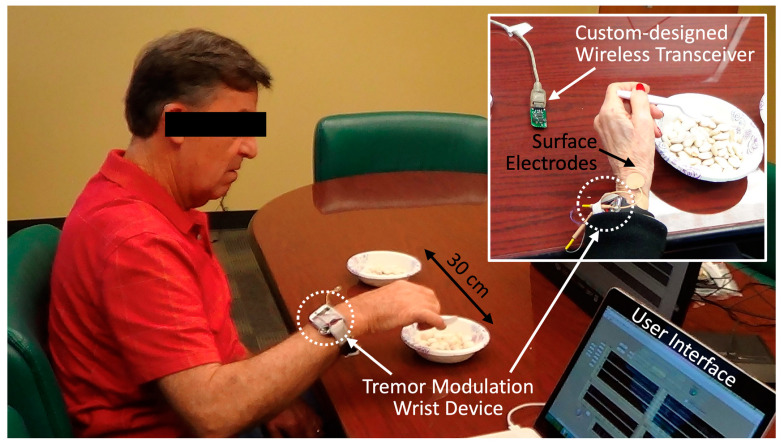
Experimental setup for peripheral nerve electrical stimulation using the wrist device while one of the participants performed the bean-transfer task.

**Figure 3:**
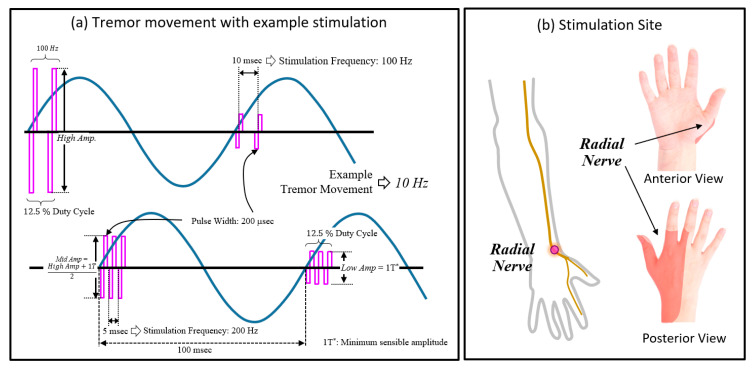
(**a**) Example electrical stimulation with tremor movement for various combinations of stimulation parameters; (**b**) stimulation site: radial nerves with their sensory nerve territories.

The overall experimental procedure is depicted in Figure 1. We began by collecting the baseline movement to analyze the dominant frequency and the peak amplitude of the subjects’ tremor when they performed a “bean-transfer” task, which involved transferring a small object (a medium-sized lima bean) from one plate to another using a spoon (Figure 2). The performance of the calibration procedure was used for the “control (no stimulation)” condition, as well as for the tremor detection algorithm. The bean-transfer task mimicked utensil movements during eating which is one of the most common and important daily activities. The subjects were asked to transfer four beans during a ten-second calibration period. Then, we placed a pair of electrodes on a branch of the designated nerve near the wrist, as shown in Figure 2. To confirm stimulation of the desired nerve branch, we asked subjects to delineate the location of stimulation-induced sensory changes. The stimulation was said to be in good position if it affected sensation in the area highlighted in Figure 3b.

Once the electrodes were properly placed, we administered electrical stimulation with two combinations of parameter settings (100 or 200 Hz frequency with 12.5% of duty cycle) to determine the minimum and maximum amplitudes of the stimulation (threshold setting). The minimum amplitude (low amp) was defined as the sensory threshold (1T), and the maximum amplitude (high amp) was defined as right below the discomfort level or ±20 V, whichever lower amplitude. The medium amplitude was defined as an average between the low and high amplitudes. The three amplitudes of stimulation (low, medium, and high) were chosen to not produce direct motoric effects (muscle twitches).

After completing the calibration and threshold setting, the participants took a five-minute break to minimize the effects of stimulation on tremor movement. Thereafter, the computer generated various combinations of electrical stimulation parameters in random order, which were applied while the participants performed the bean-transfer task. Motion sensor data were continuously collected for use in the real-time tremor detection algorithm and for later evaluation of changes in tremor characteristics under the different stimulation settings. The total number of parameter combinations was 24 (three amplitude × two frequency × four phases) for each subject. Each combination of stimulation parameters was applied in one ten-second round, and there was a ±1.5-min rest period between the rounds. We analyzed one bean-transfer as a trial; one round of stimulation consisted of four trials (n = 4 for each stimulation setting).

### 2.2. Performance Evaluation

#### 2.2.1. Human Subjects

This study was approved by the institutional review board (IRB) at Georgia Tech. We recruited nine ET participants between the ages of 47 and 82 (four males, median age 70). Written informed consent was obtained from each subject. A summary of the subject demographics is shown in Table 1. One of the subjects participated in two sessions, separated by three days. The subjects received their regular medication.

During an experimental session, we collected the subjects’ tremor movements without stimulation (baseline tremor movement), and during periods of electrical stimulation of the radial nerve, administered at combinations of stimulation parameters (Table 2). To characterize baseline tremor, we also used the score from the Essential Tremor Rating Assessment Scale (TETRAS) [52,53]. TETRAS scores were based on maximal tremor amplitudes encountered while the participants performed the bean-transfer task (TETRAS = 0: no tremor; TETRAS = 1: barely visible <1 cm; TETRAS = 2: 1 to <5 cm; TETRAS = 3: 5 to <20 cm; and TETRAS = 4: >20 cm).

#### 2.2.2. Tremor Output Metrics

We high-pass filtered (HPF) the root-mean squared three-axis accelerometer data from the wrist device with a cutoff frequency of 3 Hz and extracted tremor frequency and tremor power. We selected tremor frequency as the peak frequency of the power spectral density (PSD) of the data during the bean-transfer task. We assigned an integral of the PSD between 4 and 12 Hz in the frequency domain as the tremor power (unit: g^2^/Hz). The detailed analytical methods for the tremor output metrics appear in [47].

We defined the normalized tremor metrics as a ratio of the tremor output metrics of the control trial to those of the stimulation trial for each participant (see Equation (1)).

(1) $$\mathrm{Normalized}\;\mathrm{Tremor}\;\mathrm{Metric}\;=\;\frac{\mathrm{Tremor}\;\mathrm{Output}\;\mathrm{Metric}\;\mathrm{of}\;\mathrm{the}\;\mathrm{Stimulation}\;\mathrm{Trial}}{\mathrm{Tremor}\;\mathrm{Output}\;\mathrm{Metric}\;\mathrm{of}\;\mathrm{the}\;\mathrm{Control}\;\mathrm{Trial}}$$

If the ratio was close to 1, it equaled a control movement, and if smaller than 1, it indicated that stimulation had reduced tremor.

From a given trial with a certain stimulation parameter setting, we collected tremor movement for ten seconds and analyzed the data in four segments, each segment for one bean transfer. The average of the four segments was used to calculate the tremor metric.

#### 2.2.3. Stimulation Parameters and Combinations

**Figure 4:**
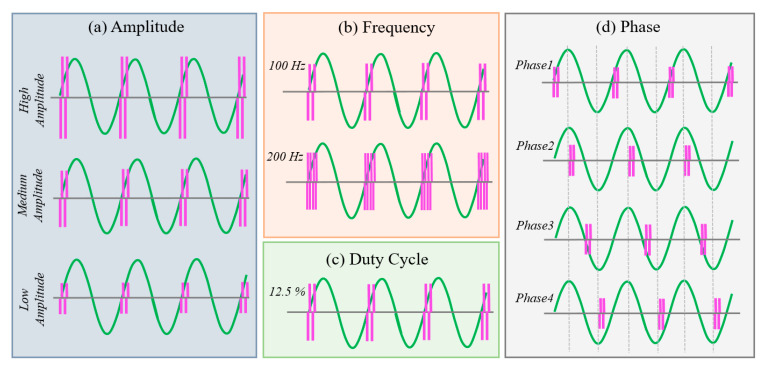
Stimulation parameters and their ranges: (**a**) amplitude, (**b**) frequency, (**c**) duty cycle, and (**d**) phase to the tremor cycle.

We used combinations of stimulation parameters, selected from three possible stimulation amplitudes, two stimulation frequencies, and phase-locked stimulation at one of four different phases of the tremor cycle. We selected combinations of stimulation parameters in random order. An example of the parameters and the combinations of parameters for the stimulation is shown in Figure 3a and Figure 4, and the stimulation parameters and their ranges are summarized in Table 2. To determine the stimulation site, we placed a pair of electrodes over a branch of the radial nerve on the wrist and adjusted the placement of the electrodes so that the subjects sensed the stimulus in the desired location (see Figure 3b, highlighted area).

### 2.3. Wearable Tremor Modulation System

**Figure 5:**
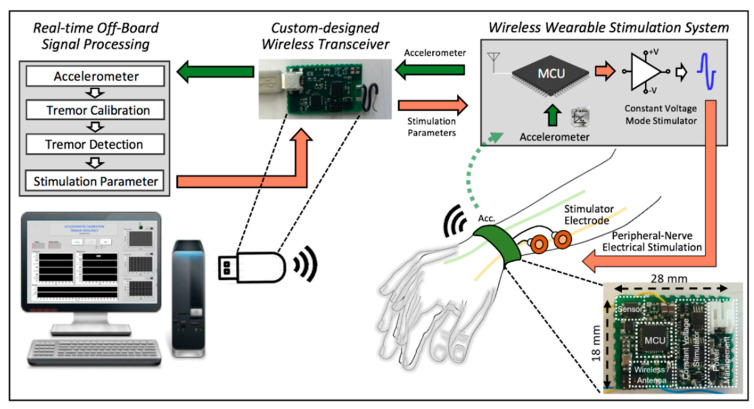
Overview of the real-time non-invasive tremor modulation system for essential tremor.

We developed a wearable tremor monitor and modulation system that includes custom-designed hardware and software (see Figure 5). We also implemented a tremor detection algorithm to be able to lock the stimulation to specific tremor phases.

#### 2.3.1. Tremor Modulation Hardware and Software

The wearable wireless real-time closed-loop stimulation system collects/analyzes tremor movements and provides voltage-mode stimulation of peripheral nerves. The system consists of a 2.4 GHz radio-frequency wireless transceiver and a pair of surface electrodes (0.8′′ round TENS Unit Electrodes, Syrtenty), and uses in its current iteration a computer with a graphical user interface (GUI) implemented on LabVIEW 2016 to help with tremor detection and modulation of parameters. In use, the electrodes are attached to the subject’s skin over the targeted peripheral nerve (such as the radial nerve), allowing us to apply trains of bipolar stimulation to the nerve. Proper choice of stimulation parameters guarantees that the stimulation does not lead to (efferent) muscle stimulation but modulates afferent components of the nerve.

The wrist device contains a motion sensor (accelerometer, LSM303D, STMicroelectronics, Plan-les-Ouates, Switzerland) and custom-designed voltage mode stimulation circuitry, controlled by a microcontroller (CC2510, Texas Instruments, Texas, USA) with a built-in 2.4 GHz RF transceiver. The electronics of the wrist device are mounted on an 18 × 28 mm^2^ printed circuit board (PCB) with an enclosure that is fastened to a commercially available wrist band. The weight of the wrist device, including the battery and enclosure, is about 36 g. The sampling rate of the motion sensor is 100 Hz. The wrist device is powered with a rechargeable 3.7 V lithium-ion battery (300 mAh) and can run up to 20 h on a single charge. A full charge cycle (via a 5 V mini-USB connector) takes about three hours. The detailed hardware and software are described in more detail in [47].

Every 10 ms, the wrist device conveys motion sensor data to a computer that runs the tremor detection algorithm, and receives, in turn, updated stimulation parameter information (stimulation amplitude, pulse width, frequency, duty cycle, phase, stimulation onset). Stimulation parameters are selected either manually or automatically. In the manual mode, the amplitude of the bi-phasic stimulus (≤20 V/phase) are applied to identify amplitude thresholds for each subject. The ranges of the parameters are summarized in Table 2. The threshold amplitudes of the minimum and maximum range had different settings. The three amplitudes of stimulation (low, medium, and high) were chosen to not produce direct motoric effects (muscle twitches).

#### 2.3.2. Tremor Detection Algorithm

We first measured the dominant frequency and peak amplitude of the patient’s tremor at baseline. We defined the “active tremor range” as tremor movements occurring at >60% of the maximal tremor amplitude and within ±30% of the dominant frequency. During the real-time “active tremor” detection mode for phase-locked stimulation, we utilized the high-pass filtered quadratic mean sensor output to apply this algorithm, and the tremor frequency was estimated by the sample differences from a five-sample moving window. Since the onset of tremor varies according to one’s movements, the stimulation phase was not constant during the trial. For phase-locked stimulation (phases 1–4), we provided stimulation when the tremor was in both the active tremor range and at the selected phase.

### 2.4. Statistical Analysis

**Figure 6:**
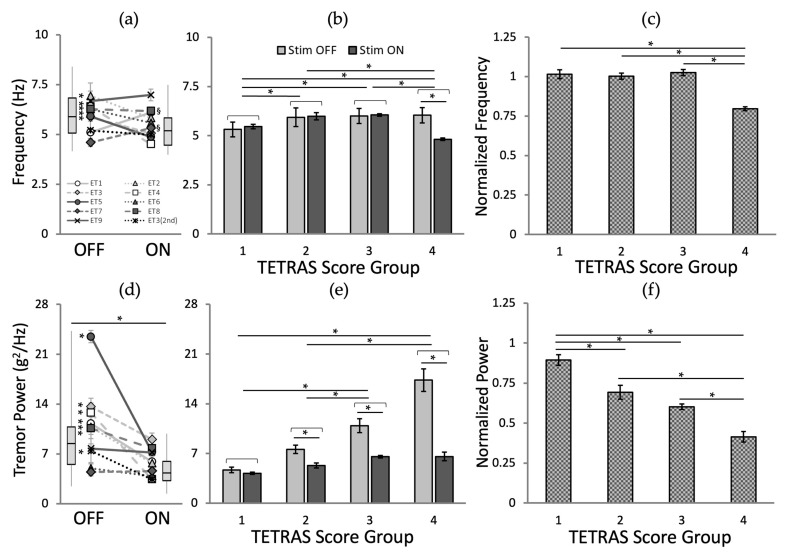
Effects of peripheral nerve stimulation. Overall tremor movement without and with stimulation for tremor metrics: dominant frequency (upper panels) and tremor power (bottom panels). The overall effect of stimulation (boxplots) with the effects of individual subjects for tremor metrics: (**a**) tremor frequency and (**d**) power. The effect of stimulation by TETRAS score groups for tremor metrics: (**b**) tremor frequency and (**e**) power. The normalized tremor metrics (metrics of stimulation/metrics of control) by TETRAS score group for tremor metrics: (**c**) tremor frequency and (**f**) power. The error bars represent standard errors of the mean. *: *p* < 0.05 for significant decrease; ^§^: *p* < 0.05 for significant increase.

To analyze the overall effects of stimulation individually (Figure 6a,d), we conducted repeated measure one-way ANOVA test (ANOVA-1; α = 0.05). To analyze the effects of normalized tremor metrics by the TETRAS score group, we conducted two-way ANOVA tests (ANOVA-2; α = 0.05) for the normalized tremor metrics. We also conducted a pairwise comparison with the least significant difference (LSD) method of the repeated measure ANOVA test to analyze the difference between the normalized metrics.

## 3. Results

### 3.1. Baseline Tremor Movements and General Effect of Stimulation

As shown in Figure 6a,d, the stimulation (across all conditions) did not alter the dominant tremor frequency across all patients (no stimulation: 5.85 ± 1.26 Hz vs. stimulation: 5.54 ± 0.95 Hz; ANOVA-1, *p* = 0.085, followed by Fisher’s LSD post hoc test), yet significantly reduced the tremor power (no stimulation: 10.93 ± 6.00 g^2^/Hz vs. stim: 5.84 ± 2.29 g^2^/Hz; ANOVA-1, *p* < 0.001, followed by Fisher’s LSD post hoc test).

Analyzed in individual cases, there was a significant decrease in five patients (* in Figure 6a), no change in three patients, and a significant increase in two (^§^ in Figure 6a). Tremor power for all but one subject (ET7) decreased with stimulation; there was a significant tremor power reduction in seven patients (* in Figure 6d).

The analysis of the dominant frequency (Figure 6b; ANOVA-2, *p* (stimulation) = 0.140, *p* (TETRAS score group) = 0.004, and *p* (stimulation ×x TETRAS score interaction) = 0.003, followed by Fisher’s LSD post hoc test) and power (Figure 6e; ANOVA-2, *p* (stimulation, TETRAS score group, and stimulation × TETRAS score interaction) < 0.001, followed by Fisher’s LSD post hoc test) was significantly affected by the TETRAS score group. The normalized tremor metrics, grouped according to baseline TETRAS groups, also suggested that there was no significant tremor frequency change except for TETRAS = 4 (Figure 6c); higher TETRAS score groups showed higher tremor power reduction ratio (Figure 6f).

### 3.2. Effects of Stimulation Amplitude

**Figure 7:**
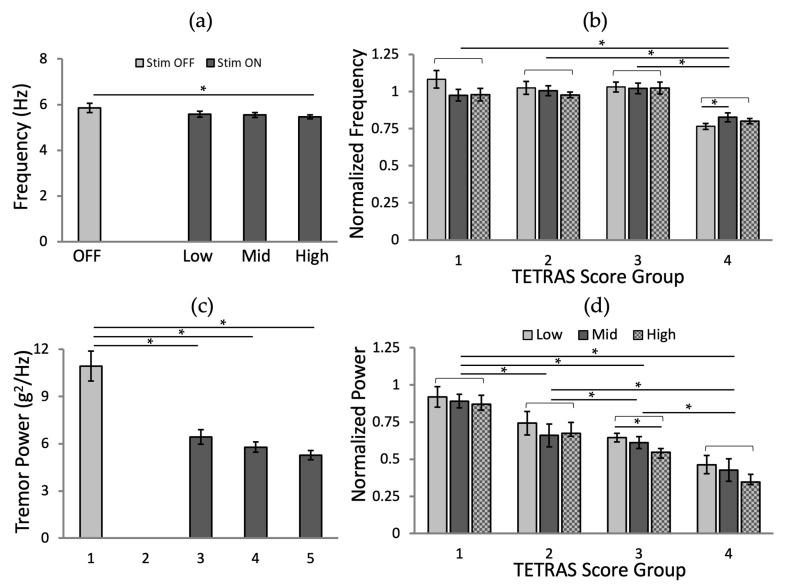
The effects of the stimulation amplitudes (low, mid, and high) by tremor metrics: (**a**) Tremor frequency and (**c**) power. The normalized tremor metrics for the different amplitudes by the TETRAS Score groups: (**b**) Tremor frequency and (**d**) power. The error bars represent the standard errors. *: *p* < 0.05.

We did not see a significant reduction in tremor frequency between the no stimulation condition and the conditions using two different amplitudes of stimulation with all frequency and phases (Figure 7a; *p* = 0.134, *p* = 0.086, and *p* = 0.036 for low, mid, and high, respectively), while tremor power was significantly reduced (Figure 7c; *p* < 0.001 for all three amplitudes). Higher amplitudes of stimulation resulted in a greater reduction rate of tremor power (Figure 7c).

We carried out an analysis of the effects of stimulation amplitude and TETRAS score group for the normalized frequency and power values. The analysis of the normalized tremor frequency (Figure 7b; ANOVA-2, *p* (amplitude) = 0.357, *p* (TETRAS score group) < 0.001, and *p* (amplitude × TETRAS score interaction) = 0.019, followed by Fisher’s LSD post hoc test) and tremor power (Figure 7d; ANOVA-2, *p* (amplitude, TETRAS score group, and amplitude × TETRAS score interaction) < 0.001, followed by Fisher’s LSD post hoc test), grouped according to pre-stimulation TETRAS scores, confirmed that there was a significant difference between the tremor amplitude and the TETRAS score interaction. There was no significant difference between the effects of different stimulation amplitudes when compared between TETRAS groups, except for TETRAS = 4, where a significant difference between the low and mid amplitudes was found (*p* = 0.046; Figure 7b); the normalized tremor power, grouped according to TETRAS scores (Figure 7d) was not differentially influenced by stimulation amplitude, except for TETRAS = 3 which showed a significant reduction at the highest stimulation amplitude (*p* = 0.027).

Based on these findings, we conclude that the stimulation does not appear to alter tremor frequency but has substantial effects on tremor power. It appears that the baseline tremor power is an important determinant of the overall success of stimulation.

### 3.3. Effects of Stimulation Frequency

**Figure 8:**
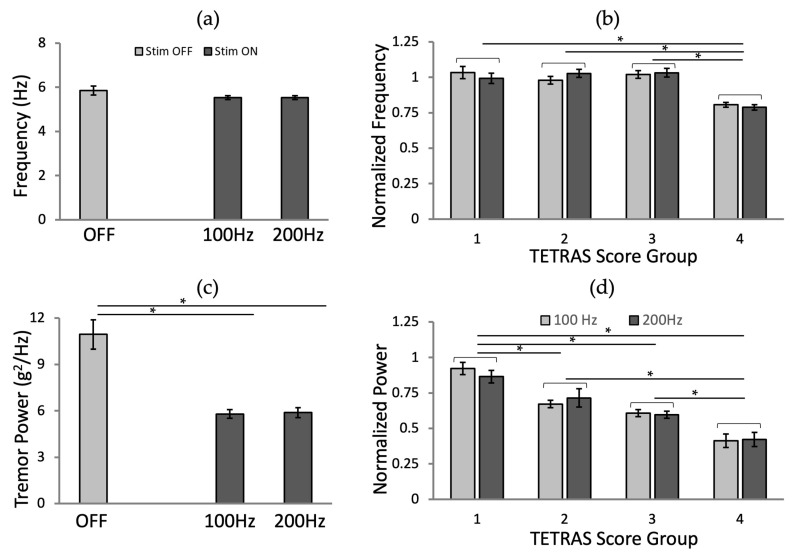
The effects of the stimulation frequency (100 and 200 Hz) by tremor metrics: (**a**) Tremor frequency and (**c**) power. The normalized tremor metrics for the different frequency by the TETRAS score groups: (**b**) Tremor frequency and (**d**) power. The error bars represent the standard errors. *: *p* < 0.05.

We found no significant effect of the frequency of stimulation on tremor frequency (Figure 8a; *p* = 0.062 and *p* = 0.064 for 100 and 200 Hz, respectively). Tremor power was significantly reduced by stimulation at either frequency (Figure 8c; *p* < 0.001 for both frequencies), with no difference between 100 and 200 Hz stimulation.

We analyzed the effects of stimulation frequency and TETRAS score group for the normalized frequency and power values. The analysis of the normalized tremor frequency (Figure 8b; ANOVA-2, *p* (frequency) = 0.991, *p* (TETRAS score group) < 0.001, and *p* (frequency × TETRAS score interaction) = 0.484, followed by Fisher’s LSD post hoc test) and tremor power (Figure 8d; ANOVA-2, *p* (frequency) = 0.885, *p* (TETRAS score group) < 0.001, and *p* (frequency × TETRAS score interaction) = 0.809, followed by Fisher’s LSD post hoc test), grouped according to pre-stimulation TETRAS scores, confirmed that there was no significant difference between the stimulation frequencies and the interaction between frequencies and TETRAS scores.

### 3.4. Effects of Stimulation Phases

**Figure 9:**
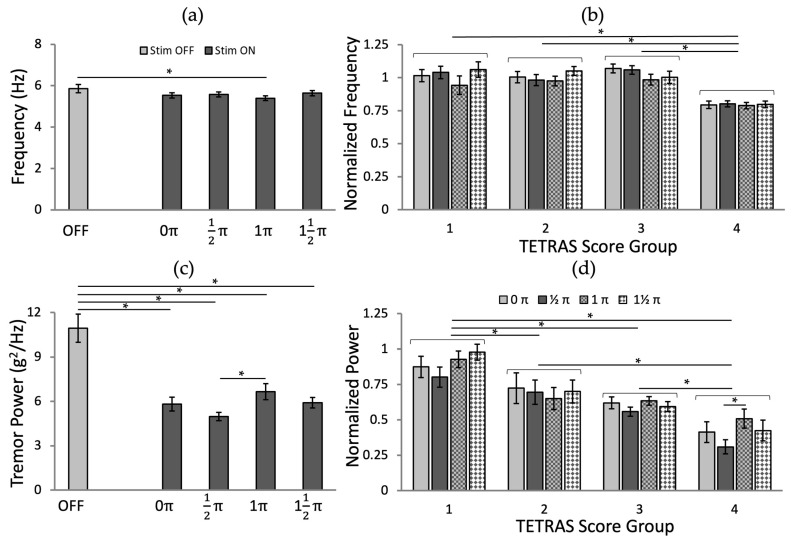
The effects of the phase-locked stimulation at four different phases by tremor metrics: (**a**) Tremor frequency and (**c**) power. The normalized tremor metrics for the different phases of stimulation by the TETRAS Score groups: (**b**) Tremor frequency and (**d**) power. The error bars represent the standard errors. *: *p* < 0.05.

The tremor frequency was not altered by stimulation, regardless of the target tremor cycle phase, except for the phase at 1 π (*p* = 0.014; Figure 9a). However, the stimulation effects on tremor power differed between groups of data with stimulation in different tremor phases (Figure 9c; *p* < 0.001 for all phases).

We also analyzed the effects of stimulation phase and TETRAS score group for the normalized frequency and power values. The analysis of the normalized tremor frequency (Figure 9b; ANOVA-2, *p* (phase) = 0.182, *p* (TETRAS score group) < 0.001, and *p* (phase × TETRAS score interaction) = 0.103, followed by Fisher’s LSD post hoc test) and tremor power (Figure 9d; ANOVA-2, *p* (phase, TETRAS score group, phase × TETRAS score interaction) < 0.001, followed by Fisher’s LSD post hoc test), grouped according to pre-stimulation TETRAS scores, confirmed that there were significant effects by the interaction between phases and TETRAS score groups for the normalized powers. From the pairwise comparison, the difference between ½ π and 1 π was significant (*p* = 0.039) for TETRAS = 4, and the phase at ½ π showed higher tremor power reduction for TETRAS = 1, 3, and 4 (Figure 9d). The TETRAS = 3 and 4 groups of records showed the most significant reduction in normalized power tremor with stimulation at phase ½ π (Figure 9d).

## 4. Discussion

Following up on our prior study of the effects of external stimulation on tremor [47], we explored the effect of stimulation amplitude, frequency, and tremor phase locking on tremor characteristics. We found that the tremor frequency remained unchanged by the stimulation, but that stimulation amplitude and phase influenced tremor power.

We found that the stimulation strongly affected tremor amplitude and frequency for the TETRAS = 4 group, and that tremor was more strongly reduced when the stimulation was applied out-of-phase in this group. The small sample size precludes meaningful analysis of this interesting finding. However, this should obviously be a topic of scientific and practical interest for a larger follow-up study.

Most published accounts of efforts to use sub-threshold electrical stimulation of afferent pathway to treat tremor [36,37,38,39,40,41,42,43,44,45,46,47,48,49,50] utilized a bulky (desktop) stimulation setup and showed tremor reductions of 14–60%. A few other studies introduced wearable setups that applied electrical stimulation [41,43,51]. While these studies included a larger number of subjects, the effects of stimulation were only studied using constant stimulation parameters. Unlike the published studies, we adapted the stimulation to the tremor cycle in real-time and found significant benefits (up to 60% tremor reduction) with out-of-phase stimulation in TETRAS = 4 patients. This finding supports the notion that tremor can be significantly reduced by out-of-phase sub-threshold stimulation [41,43], and suggests that the effects of stimulation can be optimized by adjusting stimulation parameters to ongoing tremor.

In this study, we evaluated the efficacy of the peripheral-nerve electrical stimulation with tremor metrics (dominant frequency and amplitude) using an accelerometer. However, stimulation-induced change in tremor metrics does not necessarily indicate a performance improvement for this task. This will be an important assessment in subsequent studies, aimed at investigating the practical usefulness of the device. Such assessments can be obtained using patient questionnaires, as done in our previous work [47]. Likewise, quantitative evaluations using a computer-based Fitts’ law task [54,55,56,57] to evaluate the effects of stimulation on performance will be part of such studies.

This study was a small pilot experiment and had several limitations. It focused only on the effects of radial nerve stimulation. We may have noticed other effects of the stimulation parameters on tremor if we had applied stimulation to different sites (e.g., median and ulnar nerves). In addition, we analyzed results from only nine subjects. Even though we conducted multiple trials for each parameter combination (n = 24 × 4 for each participant), the number of sessions for each TETRAS group was only 2–3. In future studies, it will be important to examine the effect of peripheral nerve stimulation on other sites and recruit more subjects and/or plan more sessions so that our conclusions are stronger. Larger group sizes may also allow additional subgroup analyses (for example, groups of different ages or genders).

Finally, even when acquired in the same patient, tremor measurements can substantially differ between sessions, perhaps depending on time of day, fatigue level, or medication states. While this variability limits the generalizability of the current study, it is also a strong argument in favor of continued development of a personalized and self-updating system to optimize stimulation effects on tremor in the future.

## 5. Conclusions

In this study, we investigated the effects of ranges of stimulation parameters in real-time phase-locked tremor modulation at the radial nerve. To evaluate the effect of the stimulation parameters, we collected motion sensor data of tremor movement with combinations of stimulation parameters compared to no stimulation and analyzed changes in dominant tremor frequency and tremor power. Combinations of the stimulation parameters consisted of the following range of parameters: amplitude (low, medium, high), frequency (100, 200 Hz), and phase (phase-locked stimulation at 0, ½, 1, 1½ π to tremor cycle). Although the stimulation frequency had a minor impact on tremor modulation, we concluded that (1) the stronger tremor group had a greater reduction in tremor power with a higher amplitude of stimulation, and (2) out-of-phase (at the ±peak of tremor) phase-locked stimulation showed a greater reduction in tremor power in groups with more severe tremor. Particularly, we found that the effects of stimulation parameters on each subject depended on the current status of the tremor amplitude, so a real-time personalized optimization model for tremor modulation would help to increase the efficacy of tremor modulation. Despite the fact that the results of this study were based on a small number of participants and sessions, we strongly believe the findings have laid a solid foundation on which to optimize stimulation parameters, maximize the effects of tremor reduction, and minimize nerve fatigue and power consumption for consistent, long-term effects; ultimately, a promising alternative personalized treatment to existing Parkinson’s Disease and related disorders treatment.

## Figures and Tables

**Table 1:** Summary of subject demographics.

Subject Demographics	n (%)	Mean (SD)	Range
Age	–	67.67 (11.67)	47–82
Gender, male/female	4 (44%)/5 (56%)	–	
Years since diagnosis	–	16.33 (14.59)	4–50
Device placement, right	7 (77.78%)	–	–
Medication	5 (56%)	–	–
TETRAS (1–4)	–	2.70 (1.16)	1–4
Dominant frequency (Hz)	–	5.86 (1.26)	3.60–9.20
Tremor power (g^2^/Hz)	–	10.33(6.00)	3.60–24.94

**Table 2:** Stimulation parameters and their ranges.

**Stimulation Parameters**	**Settings Used in This Study**
Amplitude	Low = 1T, Medium = $\frac{\mathrm{Low}+\mathrm{Max}}{2}$, High = Max
Pulse width	200 µs
Frequency	100 Hz, 200 Hz
Duty cycle	12.5%
Phase	Phase-locked to the tremor cycle at 0 π, ½ π, 1 π, and 1½ π (Phase 1, 2, 3, or 4, respectively).

T = Sensory thresholds.
